# Informal caregivers of people living with dementia as invisible second patients: examining the dual role of burden and resilience in perceived stress

**DOI:** 10.1186/s12877-026-07974-x

**Published:** 2026-07-14

**Authors:** Emre Özbulut, Alexandra Wuttke, Katharina Geschke, Erik Farin-Glattacker

**Affiliations:** 1 Section of Health Care Research and Rehabilitation Research, Institute of Medical Biometry and Statistics, Medical Faculty and Medical Center - University of Freiburg, Freiburg, Germany; 2 Department of Psychology, Clinical Psychology and Psychotherapy of Older Age, University of Konstanz, Konstanz, Germany; 3 Department of Psychiatry and Psychotherapy, University Medical Center of the Johannes Gutenberg University Mainz, University Medical Center Mainz, Mainz, Germany

**Keywords:** Dementia, Informal caregivers, Stress, Caregiver burden, Caregiver resilience

## Abstract

**Background:**

Informal caregivers (ICs) of people living with dementia (PwD) are often referred to as invisible second patients, as they frequently experience chronic stress and related health issues. Identifying factors that contribute to or buffer caregiver stress is therefore of critical importance. This study aimed to explore how caregiver burden and resilience jointly are associated with stress among ICs of PwD – a rarely adopted approach.

**Methods:**

The sample consists of *n* = 172 ICs. To examine the associations between our dependent variable, stress (PSS), and caregiver burden (BIZA-D-PV), caregiver resilience (BRS), the quality of life of the PwD (QoL-AD), as well as the IC’s sociodemographic characteristics, we conducted a hierarchical multivariate regression analysis. Resilience was included as a second block to assess changes in model fit and variable effects. Furthermore, an interaction model was calculated to investigate potential moderating effect of resilience.

**Results:**

In Block 1, without considering resilience, personal constraints of the IC (*p* < 0.001), the QoL of the PwD (*p* < 0.001), and cohabitation of the IC and PwD (*p =* 0.006) were significantly associated with stress. In Block 2, only personal constraints (*p* < 0.001) and the QoL of the PwD (*p =* 0.001) remained significant, and, additionally, the explained variance (*R²*) increased significantly (*p* < 0.001). In the interaction model, only the main effects of resilience (*p =* 0.004), personal constraints (*p =* 0.002), and QoL of the PwD (*p* = 0.002) were significant.

**Conclusions:**

These findings might highlight important considerations for future interventions aimed at supporting ICs and PwD. Special attention could be given to cohabiting IC-PwD dyads, as these relationships often involve unique challenges and stressors that could be mitigated through tailored support strategies.

**Trial registration:**

German Register of Clinical Trials (Deutsches Register Klinischer Studien, DRKS), DRKS00023560. Registered 13 November 2020 Retrospectively registered, https//www.drks.de/drks_web/navigate.do? navigationId=trial.HTML&TRIAL_ID=DRKS00023560.

## Introduction

### Background

Informal caregivers (ICs) of people living with dementia (PwD) are often referred to as invisible second patients [1], as they frequently experience chronic stress, making them particularly vulnerable to the development of stress-related psychological and physical health issues [2]. After all, the negative health consequences of stress are well known [3]. Overall, a majority of ICs are exposed to high levels of stress [4]. Depression is more prevalent in ICs than in non-caregivers [5, 6]. From a dyadic point of view, PwD and their caregivers influence each other; particularly when ICs are chronically stressed, emergency hospital admissions of PwD occur more often because of the exhaustion of ICs [6]. Thus, understanding which factors drive and which factors buffer stress is highly relevant, as the stability of care for PwD is highly dependent on the stress level and thus health of the IC [7].

In understanding stress in IC, caregiver burden plays an important role. Whereas acute stress is adaptive, it is chronic stress that leads to adverse health effects [8]. Chronic stress is associated with higher amounts of caregiver burden. There is a plethora of studies investigating caregiver burden. However, it is not yet clear how these care-related burden factors affect stress. It is important to investigate and distinguish these factors, as stress is ultimately one of the primary causes of the associated negative health outcomes. There are manifold factors known from the literature that influence caregiver burden. These can be grouped into three categories: factors associated with the IC, factors associated with the PwD and factors associated with the social context. First, dementia care is often extensive, intensive, and challenging. As a result, social burden or the time spent on care tasks can represent significant stressors [9]. Furthermore, personal constraints arise from the fact that, owing to the degree or duration of care, the needs and leisure time of the IC are often neglected [10, 11]. Gottschalk et al. [12] reported that reaching a balance between “care and personal needs is often challenging.” Moreover, cohabitating with PwD in the same household [13] as well as the (female) gender of the caregiver can contribute to stress or mental health problems for the IC [11]. Second, the degree of cognitive impairment and dementia-specific behavioral disturbances (BPSDs), such as aggressiveness or disoriented behavior, may be significant stressors for ICs [14–16]. Additionally, it is well-documented that, given the dyadic nature of coping in dementia, the PwDs quality of life is associated with the ICs perceived stress [17]. Third, in addition to coping strategies that are effectively supported by social support [15], the psychological well-being of ICs is also dependent on the level of support they receive from their social environment [18, 19]. Although there is more evidence regarding caregiver burden and informal or formal support [11, 20], this suggests that a missing social appreciation (e.g., through sympathy from the ICs environment) may also be associated with increased stress for ICs.

From a broader theoretical perspective, the stress-process model proposed by Pearlin et al. [21] suggests that caregiver stress is shaped by a range of factors, including sociodemographic background, primary caregiving demands, secondary role-related strains, and protective resources such as social support and coping strategies. In this context, resilience may also represent an important protective resource associated with ICs stress experiences. However, while many studies have examined the factors associated with caregiver burden, knowledge about protective factors related to stress in IC lags behind. The majority of caregivers experience stress and caregiver burden. At the same time, however, the majority of ICs remain healthy. Resilience is defined as the ability to maintain health despite adversity. Recent research has identified factors associated with caregiver resilience. For example, higher levels of resilience are linked to better coping skills, improved mental health, and higher quality of life [22]. Palacio et al. [23] highlighted an association between resilience and reduced emotional distress, as well as the significant role of social support in the development of resilient coping strategies. Moreover, caregivers who live together with patients (cohabitation) appear to be less resilient [20]. Some of the main psychological factors associated with caregiver resilience are not only stress but also caregiver burden [24]. One might argue that, as a “counterpart” of the numerous factors contributing to caregiver burden (mentioned earlier in this study), caregiver resilience can have a positive effect on IC burden or stress [23, 25, 26]: “Resilience is a multifaceted response to the caregiving role and is influenced by a multitude of interrelated factors” [20].

So far, a substantial body of empirical evidence has been generated on individual factors on the burden side as well as on the resilience side. However, what is still largely missing is an overarching, care-specific model that integrates these findings into a coherent framework tailored to caregiving contexts. While general stress and resilience models can offer useful starting points, the specificity of the caregiver situation cannot be adequately captured within generic stress, coping, and resilience frameworks. For this reason, Wuttke-Linnemann et al. [7] advocated considering both burden and resilience simultaneously to understand their impact on caregiver health and stress, whereas to date, most studies have focused either on caregiver burden or caregiver resilience in isolation. They introduce a scale that weights burden and resilience factors against each other to identify ICs at particular risk for adverse health outcomes [27, 28]. Following this rationale, informal caregiving is not defined by burden alone; the likelihood of adverse caregiver outcomes depends on the balance between caregiving-related stressors (burden) and resilience-related resources (protective factors). Drawing on a homeostatic/allostatic perspective, sustained strain that is insufficiently buffered by resilience resources increases vulnerability, whereas adequate psychological and social resources can offset comparable levels of stress. The ResQ-Care was therefore designed to assess *both* domains within one instrument and to express their relationship as a stress-to-resilience ratio. This ratio provides an interpretable index of whether demands currently outweigh resources, supports risk stratification, and offers a direct clinical implication: interventions can be targeted either at reducing strain, strengthening resilience resources, or both, depending on the individual profile. The scale is subject to ongoing empirical investigations and may provide additional explanatory value for understanding IC stress by integrating burden and resilience factors simultaneously.

### Objectives and hypotheses

The primary objective of this study was to examine exploratorily how caregiver burden and resilience jointly relate to stress among ICs of PwD. Although these constructs are typically examined separately, their joint contribution remains largely unexplored. Based on well-documented empirical relationships, we propose the following hypotheses (please note hypotheses 1 and 2 can be considered replicational/confirmatory as they built on well-documented associations; whereas hypotheses 3 focuses on the additional value of the joint consideration of burden and resilience).: (1) Caregiver burden is positively associated with stress in ICs; (2) Caregiver resilience shows a negative association with stress under control of caregiver burden.; (3) Higher resilience weakens (attenuates) the positive effect of burden on stress.

## Methods

### Study design & participants

The data of this study were collected as part of our prospective, randomized controlled intervention study to optimize care for PwD and ICs, DemStepCare. The DemStepCare trial evaluated a dementia-care model in which general practitioners received multidisciplinary support – including training, case management, risk assessment, electronic records, medication review and connections to regional services – to address the individual needs of people with dementia and their caregivers. The intervention was compared with usual care to assess its impact on patients’ quality of life, caregiver burden and hospital utilization (*further details are provided in the study protocol* [29])*.* The study received approval from the Ethics Committee (Reference number: 2019–14427). The sample included ICs of PwD who were enrolled in the study through their general practitioner. Each PwD was cared for by a maximum of one IC, and no IC cared for more than one PwD. The inclusion criteria for PwD were a confirmed dementia diagnosis, a private residence (nursing home = exclusion), and living within the study area (Rhineland-Palatinate, predominantly rural structures). The ICs received a questionnaire if they were officially listed as a relative in the DemStepCare-study. The number of participants who returned their IC questionnaire was *n* = 172. All data in this examination are based on baseline measurements taken between 2019 and 2022 and before the intervention. All analyzes in this paper are cross-sectional and are conducted with Stata 14.

### Measures

#### Stress

We measured IC stress via the validated German version of the Perceived Stress Scale (PSS) [3]. These instruments ask participants about their feelings and thoughts over the past month, specifically how often they experienced the stressful situations described in the 10 items were (range = 1 (never) to 5 (always)). For example, ICs had to answer the following questions: “In the last month, how often have you felt nervous and ‚stressed‘?“ and “In the last month, how often have you found that you could not cope with all the things that you had to do?”. The PSS score is calculated by summing the item responses (ranging from 10 (low stress) to 50 (high stress)). With the PSS, we are able to measure the perception of (chronic) not care-related stress. To account for the validity or, respectively, specific characteristics of our sample, Cronbach’s alpha (*α* = 0.88) was calculated using our data (as with all subsequent Cronbach’s *α* values).

#### Caregiver burden

The Berlin Inventory on Relatives’ Burden in Dementia (Berliner Inventar zur Angehörigenbelastung-Demenz-Praxisversion, BIZA-D-PV) is validated and specifically designed to assess burden factors over the past four weeks, focusing on “care-related demands and psychosocial impairments arising from care [for the relative]” [30]. The practice version (PV) consists of six modules. For this study, we included the following five scales (29 questions) as individual variables:

1. “Burden due to cognitive impairment” (range = 0 (not at all) to 16 (strongly), Cronbach’s *α* = 0.87),
2. “Burden due to aggression and disoriented behavior” (range = 0 (not at all) to 16 (strongly), Cronbach’s *α* = 0.72),
3. “Burden due to personal constraints” (range = 0 (never) to 16 (always), Cronbach’s *α* = 0.95),
4. “Burden due to care tasks” (range = 0 (someone else provides all of the support) to 36 (I provide all of the support), Cronbach’s *α* = 0.91),
5. “Burden due to missing social appreciation” (range = 0 (never) to 20 (always), Cronbach’s *α* = 0.87).

Each of these modules reflects a subscale, with Likert-scaled item responses summed to create scores.

#### Resilience

The Brief Resilience Scale (BRS) measures “the ability to bounce back or recover from stress” (without specifying a temporary condition) [31]. For instance, the validated German version of the BRS includes items such as “I tend to bounce back quickly after hard times” and “I have a hard time making it through stressful events.” Overall, the questionnaire consists of six items (Cronbach’s *α* = 0.75) and calculates a mean value for statistical analysis (range = 1 (strongly disagree) to 5 (strongly agree)) [32].

#### Quality of Life and sociodemographic characteristics

Due to the well‑documented association with caregiver stress, the PwDs quality of life (QoL) was included as a covariate in the regression analyses. We operationalized the QoL of PwD via the validated Quality of Life in Alzheimer’s Disease (QoL-AD) scale. Notably, this is the proxy version, as the IC provided the information. The QoL-AD is a concise instrument with 13 items (Cronbach’s *α* = 0.83) that assess the QoL of PwD, with a particular focus on domains relevant to cognitively impaired elderly individuals [33]. The sum score (range = 13 (poor) to 52 (excellent)) was used for further analyses.

As sociodemographic control variables, we also assessed the gender of the IC (1 = female, 2 = male, 3 = diverse (note: no participants in this dataset identified as “diverse”)), as well as cohabitation status (“Do you live with your relative in the same household?“: 1 = yes, 0 = no). The participants were also asked about their age, relationship with the patient (1 = spouse, 2 = children, 3 = other), and employment status (1 = yes, 0 = no).

### Statistical analysis

Multiple linear regressions were conducted. Given the sample size, stepwise forward variable selection was used exploratorily to identify the strongest independent variables while maintaining model parsimony; candidate variables were selected based on theoretical and empirical considerations. Additionally, only variables with a p-value threshold of < 0.20 (bivariate correlation with stress) were entered into the stepwise selection procedure to avoid excluding potentially important factors and to ensure model robustness. Furthermore, variables were not included if they caused a reduction in the number of observations by > 5%. The primary model, first, was estimated hierarchically:

- Block 1 = BIZA-subscales (burden due to care tasks, personal constraints, cognitive impairment, BPSD and missing social appreciation), QoL of the PwD and sociodemographics of the IC;
- Block 2 = All variables from block 1 with resilience adjustment.

In this way, we examined the improvement in model fit by calculating the change in *R²* and the *F*_change_ statistic between the two models [34]. We additionally examined to what extent the inclusion of resilience in the second model attenuated or eliminated the statistical significance of factors that were significant in the first model. Furthermore, a third (interaction) model was calculated to investigate the moderating effect of resilience on the relationship between burden and stress. To facilitate independent interpretation of main and interaction effects and to reduce multicollinearity, in this model, all continuous factors were mean-centered, and interaction terms were created by multiplying the centered moderator (resilience) with both the continuous as well as dummy-coded variables (cohabitation and gender of the IC). Assuming at least medium effect sizes (based on theory and according to Cohen), the statistical power (*α* = 0.05) was very good for models 1 (0.94) and 2 (0.93), and good for model 3 (0.81). For potentially smaller effects, however, the power of all three models would not be sufficient.

## Results

### Participants

Table 1 presents detailed descriptive statistics for the clients with dementia and the study participants. Our sample consisted of *n* = 172 ICs. The mean age of the ICs was 65.6 years. The PwD in our sample had a mean age of 81.4 years and were almost equally likely to be cared for by spouses (42.4%) or (biological) children (45.4%). Moreover, the majority of participants were not employed or were retired (50%). In terms of sex distribution, the proportion of female ICs was greater (67.4%).

**Table 1:** Sample descriptive statistics (*n* = 172)

Variable	*N* (%)	Mean (SD)	Range
Age of the PwD		81.4 (7.2)	56–99
Gender of the PwD			
Female	102 (59.3%)		
Male	66 (38.4%)		
Missing	4 (2.3%)		
Age of the IC		65.59 (11.8)	32–87
Relationship			
Spouse	73 (42.4%)		
Children	78 (45.4%)		
Other	12 (7.0%)		
Missing	9 (5.2%)		
Gender of the IC			
Female	116 (67.5%)		
Male	47 (27.3%)		
Missing	9 (5.2%)		
Employment			
Yes	65 (37.8%)		
No	86 (50.0%)		
Missing	21 (12.2%)		
Cohabitation			
Yes	96 (55.8%)		
No	71 (41.3%)		
Missing	5 (2.9%)		
Stress (PSS)		28.65 (7.2)	11–47
Resilience (BRS)		3.26 (0.7)	1.33–5.0
Burden due to personal constraints (BIZA-D)		8.31 (5.9)	0–20
Burden due to BPSD (BIZA-D)		5.27 (4.8)	0–20
Burden due to cognitive impairment (BIZA-D)		8.00 (4.7)	0–16
Burden due to missing social appreciation (BIZA-D)		7.23 (5.1)	0–20
Burden due to care tasks (BIZA-D)		12.21 (10.9)	0–36
Quality of Life of PwD – Proxy (QoL-AD)		28.30 (5.7)	16–50.8

*Abbreviations*: *BIZA-D* Berlin Inventory on Relatives’ Burden in Dementia,* BPSD* Dementia-specific behavioral disturbances, *BRS* Brief Resilience Scale, *IC* informal caregivers,* N* Number of participants,* PSS* Perceived Stress Scale, *PwD* People living with dementia, *QoL-AD* Quality of Life in Alzheimer’s Disease, *SD* Standard deviation

Regarding the variables measured, the mean ICs stress level was moderate (28.65). The same applies to caregiver resilience, the QoL of the PwD, and the burden related to personal constraints and cognitive impairments. The means for burden due to BPSD, care tasks, and missing social appreciation were in the lower ranges of their respective scales, suggesting that, on average, the perceived burden related to these factors was low.

### Regression analysis

Tables 2 and 3 show the main results of the two models or blocks (without and with resilience).

**Table 2:** Hierarchical regression model: block 1

Stress (PSS)	Coef. (Ustd.)	Std. Err.	t	*p*	[95% CI]	
Personal Constraints (BIZA-D)	0.485	0.125	3.88	< 0.001***	0.238	0.732
BPSD (BIZA-D)	0.038	0.134	0.28	0.779	-0.228	0.303
Cognitive Impairment (BIZA-D)	-0.016	0.135	-0.12	0.905	-0.284	0.251
Missing Social Appreciation (BIZA-D)	0.208	0.125	1.67	0.098	-0.039	0.454
Care Tasks (BIZA-D)	-0.082	0.055	-1.49	0.139	-0.191	0.027
Quality of Life – Proxy (QoL-AD)	-0.320	0.103	-3.10	0.002**	-0.523	-0.116
Cohabitation (yes)	3.123	1.128	2.77	0.006**	0.892	5.353
IC Gender (female)	1.065	1.092	-0.98	0.331	-3.223	1.093
Model statistics: adjusted *R²* = 0.38, *p* < 0.001, 95% CI, *N* = 150						

*Abbreviations:BIZA-D* Berlin Inventory on Relatives’ Burden in Dementia,* BPSD* Dementia-specific behavioral disturbances,* BRS* Brief Resilience Scale,* CI* Confidence interval,* Coef.* Coefficient,* IC* Informal caregivers,* p p-*value,* PSS* Perceived Stress Scale,* QoL-AD* Quality of Life in Alzheimer’s Disease,* Std. Err.* Standard error,* t* t-statistic,* Ustd.* Unstandardized

*p < 0.05, **p < 0.01, ***p < 0.001

**Table 3:** Hierarchical regression model: block 2

Stress (PSS)	Coef. (Ustd.)	Std. Err.	t	*p*	[95% CI]	
Resilience (BRS)	-4.431	0.712	-6.22	< 0.001***	-5.839	-3.023
Personal Constraints (BIZA-D)	0.407	0.114	3.57	< 0.001***	0.182	0.632
BPSD (BIZA-D)	0.176	0.125	1.40	0.163	-0.072	0.424
Cognitive Impairment (BIZA-D)	-0.145	0.123	-1.17	0.244	-0.389	0.100
Missing Social Appreciation (BIZA-D)	0.118	0.114	1.04	0.301	-0.110	0.343
Care Tasks (BIZA-D)	-0.033	0.052	-0.64	0.526	-0.135	0.069
Quality of Life – Proxy (QoL-AD)	-0.308	0.094	-3.29	0.001**	-0.494	-0.123
Cohabitation (yes)	0.730	1.093	0.67	0.505	-1.432	2.892
IC Gender (female)	0.523	0.989	0.53	0.598	-1.432	2.478
Model statistics: adjusted *R²* = 0.51, *p* < 0.001, 95% CI, *N* = 146						

*Abbreviations*: *BIZA-D *Berlin Inventory on Relatives’ Burden in Dementia,* BPSD* Dementia-specific behavioral disturbances,* BRS* Brief Resilience Scale,* CI* Confidence interval,* Coef.* Coefficient,* IC* Informal caregivers,* p p-*value,* PSS* Perceived Stress Scale,* QoL-AD* Quality of Life in Alzheimer’s Disease,* Std. Err.* Standard error,* t* t-statistic,* Ustd.* Unstandardized

*p < 0.05, **p < 0.01, ***p < 0.001

In block 1 (without resilience, adjusted *R²* = 0.38), personal constraints of the IC were positively, and with a moderate to strong effect, associated with ICs stress (PSS) as our outcome variable (β = 0.38, *p* < 0.001). Additionally, a decrease in the QoL of the PwD was significantly associated with a higher perceived stress level (β = -0.32, *p* = 0.002). Cohabitation with the PwD was also associated with a significant increase in IC stress (β = 0.21, *p* = 0.006). All other factors were not significant.

In block 2 (with resilience, adjusted *R²* = 0.51), the inclusion of resilience increased the explained variance in *R²* by approximately 13%, suggesting a moderate incremental contribution of the factor according to Cohan’s conventions, and the change in the F value between the two models was highly significant (*p* < 0.001). Caregiver resilience moderately to strongly and significantly decreased stress in IC (β = -0.41, *p <* 0.001).

The inclusion of resilience (block 2) also influenced the significance of the other factors. In this second model, in addition to the variables that were already insignificant in the first model, the effect of cohabitation on stress was no longer significant. However, personal constraints (β = 0.31, *p* < 0.001) and the QoL of the PwD (β = -0.23, *p* = 0.001) remained significant, with their direction unchanged. Although no other variables changed from significant to insignificant (or vice versa) between the two models, the significance levels of the caregiver burden variables were notably influenced, with resilience having a highly significant and large effect on IC stress.

No relevant violations of regression assumptions were observed in either model: Statistically relevant multicollinearity was ruled out using the Variance Inflation Factor (VIF) (mean VIF = 1.66; all independent variables < 2.35 in both models). The Shapiro-Wilk test indicated no significant deviation from normality of the residuals in model 1 (*W* = 0.98, *p* = 0.120) and model 2 (*W* = 0.99, *p* = 0.438). The Breusch-Pagan/Cook-Weisberg tests indicated no evidence of heteroskedasticity in either model (Model 1: *χ²*(1) = 0.04, *p* = 0.848; Model 2: *χ²*(1) = 0.01, *p* = 0.907).

In the third model, no significant effects of the interaction terms were observed (see Table 4). Only the main effects of resilience (β = -0.50, *p* = 0.004), personal constraints (β = 0.29, *p* = 0.002), and QoL (β = -0.23, *p* = 0.002) were significant – representing medium to large effect sizes. The model again demonstrated good explanatory power (adjusted *R²* = 0.50, *p* < 0.001). Regression diagnostics indicated no evidence of multicollinearity (mean VIF = 2.65; all VIFs < 10.00), significant violation from normality of the residuals (*W* = 0.99, *p* = 0.495), or heteroscedasticity (*χ²*(1) = 0.38, *p* = 0.537).

**Table 4:** Interaction model (all independent variables mean-centered)

Stress (PSS)	Coef. (Ustd.)	Std. Err.	t	*p*	[95% CI]	
Resilience (BRS)	-5.488	1.893	-2.90	0.004**	-9.233	-1.743
Personal Constraints (BIZA-D)	0.372	0.118	3.16	0.002**	0.139	0.607
BPSD (BIZA-D)	0.209	0.131	1.60	0.111	-0.049	0.467
Cognitive Impairment (BIZA-D)	-0.135	0.127	-1.06	0.293	-0.387	0.118
Missing Social Appreciation (BIZA-D)	0.135	0.117	1.15	0.253	-0.097	0.367
Care Tasks (BIZA-D)	-0.036	0.054	-0.66	0.508	-0.143	0.071
Quality of Life – Proxy (QoL-AD)	-0.305	0.096	-3.18	0.002**	-0.495	-0.115
Cohabitation (yes)	0.815	1.141	0.71	0.477	-1.444	3.074
IC Gender (female)	0.700	1.038	0.67	0.502	-1.346	2.754
Personal Constraints *x* BRS	0.308	0.183	1.68	0.096	-0.056	0.671
BPSD *x* BRS	-0.083	0.221	-0.37	0.709	-0.520	0.354
Cognitive Impairment *x* BRS	-0.211	0.179	-1.18	0.240	-0.565	0.143
Missing Social Appreciation *x* BRS	0.087	0.160	0.54	0.588	-0.229	0.402
Care Tasks *x* BRS	-0.104	0.080	-1.30	0.195	-0.262	0.054
Quality of Life – Proxy *x* BRS	0.031	0.142	0.22	0.826	-0.249	0.312
Cohabitation (yes) *x* BRS	0.494	1.684	0.29	0.770	-2.838	3.826
IC Gender (female) *x* BRS	0.796	1.779	0.45	0.655	-2.723	4.316
Model statistics: adjusted *R²* = 0.50, *p* < 0.001, 95% CI, *N* = 146, *x* = interaction with BRS						

*Abbreviations:BIZA-D* Berlin Inventory on Relatives’ Burden in Dementia,* BPSD* Dementia-specific behavioral disturbances,* BRS* Brief Resilience Scale, *CI* Confidence interval, *Coef.* Coefficient,* IC* Informal caregivers* p p-*value, *PSS* Perceived Stress Scale,* QoL-AD* Quality of Life in Alzheimer’s Disease, *Std. Err.* Standard error,* t* t-statistic,* Ustd.* Unstandardized

*p< 0.05, **p < 0.01, ***p < 0.001

## Discussion

The present study examined the simultaneous associations of burden and resilience with stress in ICs of PwD. Overall, higher burden was linked to higher stress levels, whereas higher resilience dispositions were associated with lower stress levels. In addition, resilience showed a moderate incremental effect beyond burden-related variables. These findings are broadly consistent with the stress-process model proposed by Pearlin et al. [21], which conceptualizes caregiver stress as the result of interacting demands, strains, and protective resources. Our findings may also offer starting points for the development of interventions that simultaneously target burden and resilience in a more comprehensive and preventive manner. In our sample, personal constraints experienced by the IC were strongly associated with stress, whereas resilience was significantly linked to lower stress. Furthermore, a low QoL of the PwD was found to be associated significantly with high stress. These three factors account for a substantial portion of the variance in the regression models with the outcome variable stress (which confirms hypotheses 1 and 2.). One of the key findings of this study is that personal constraints and the QoL of PwD seem to have a significant effect on stress, even when the strong effect of resilience is controlled for or resilience moderated their relationship with stress (which at least partly falsifies hypothesis 3). These findings suggest that interventions targeting these factors should be prioritized in the care of PwD and their ICs although it is known that their acceptance is rather low in ICs of PwD [35]. Personal constraints (e.g. limited opportunities for personal retreat, insufficient time to care for one´s own health, or reduced time for social activities) might be mitigated by increasing support for ICs and PwD, especially if caregivers recognize the urgency of preventive measures aimed at strengthening their own resilience. These findings emphasize the critical role that social resilience factors play in caregiver health. Moreover, the personal constraints and QoL of PwD should not be considered in an isolated manner. From a dyadic perspective, it is plausible that these factors interact at an interpersonal level, highlighting the importance of addressing both ICs and PwD together when interventions are designed to relieve caregiver burden and increase resilience.

The importance of resilience in explaining stress is well established, and our findings confirm its significant association with stress. The stability of the caregiving situation depends heavily on the individual resilience of the IC [20], and resilience should therefore be a central focus of dementia care. In regard to personal constraints, there appears to be a need to reduce the burden related to personal constraints, unattached from or in addition to existing high-resilience dispositions in IC. The same recommendation applies to improve the QoL of PwD. The low QoL of PwD, as a psychosocial construct, not only increases caregiver burden (the focus of previous studies, e.g. [36]), but also contributes directly to IC stress. Our research strengthens the limited existing evidence regarding the relevance of the QoL of PwD [11, 14]. Resilience alone cannot compensate for the negative health impact of a low QoL.

It remains unclear whether resilience influences the processing of stress by altering the perception of specific burden factors. On the one hand, our analysis suggests that individual resilience dispositions may be associated with attenuated associations between the other explanatory variables and the outcome. On the other hand, the observed attenuation of these associations was either moderate or related to burden forms that did not significantly explain IC stress anyway. Small interaction effects were observed, but none reached statistical significance. Nonetheless, there are indications in this study that resilience plays a crucial role. Therefore, resilience should be strengthened not only when personal constraints are high but also when other forms of high burden stresses IC. Resilience appears to be trainable through psychological interventions in ICs [27]. There is currently no consensus as to when an intervention is considered “resilience training” or which components are important and particularly effective but psychoeducation, mindfulness-based approaches, and cognitive behavioral therapy techniques appear to be particularly effective in promoting resilience in ICs [27].

Another important finding is the absence of significant effects from the other four burden variables. This is particularly surprising in relation to the burden caused by the BPSD of PwD, as BPSD are frequently identified as major stressors in the literature (e.g. [14–16]). At the same time, this may underline the substantial explanatory value of the three aforementioned factors. In contrast to other studies that highlights the impact of psychosocial, informal and formal support on stress [18, 19], no significant effect was observed for missing social or mental support in our sample. However, personal constraints are often a consequence of missing social appreciation, and we noted that this variable was generally low in our sample. Furthermore, intensive and challenging dementia care has been well documented as a risk factor for stress [9]. In contrast, our sample reported a low burden due to care tasks on average, which may explain why this variable does not serve as a suitable predictor of stress. Notably, the QoL of PwD is closely associated with dependence on caregivers and thus indirectly reflects the extent of caregiving tasks. One possible explanation for the absence of significant effects from the other four caregiver burden variables is the dominant relevance of resilience, personal constraints, and the QoL of the PwD in explaining stress – as with BPSD. This underscores the importance of focusing on these three factors when designing interventions to support ICs and optimize dementia care.

With respect to the cohabitation of IC and PwD, living together appears to significantly affect stress only when the resilience of the IC is not considered. This result might indicate that resilience is a more important explanatory variable of stress than cohabitation. When we simultaneously consider cohabitation and resilience, we differ from some previous studies, which found that cohabitation itself indicates a greater risk of stress or psychological overload [13]. Nevertheless, living together can be a source of stress for the IC. However, this stress may be mitigated or compensated by higher resilience. Since both the main and interaction effects were small and non-significant in this regard, this question cannot be answered based on our study. Nevertheless, ICs with low-resilience dispositions should receive support in managing the stress or burden associated with living together, which may involve a reduced sense of personal space or increased caregiving responsibilities. In contrast, the gender of the IC did not contribute significantly to explaining IC stress.

One strength of this study is the evidence it provides regarding the simultaneous associations among IC stress, caregiver burden, and caregiver resilience. We demonstrated that resilience cannot fully counterbalance all stressors and that personal constraints stand out as particularly impactful stressors, often linked to aspects such as accepting support.

However, several limitations should be noted. First, a low QoL of PwD could theoretically also be an outcome of greater IC stress; the direction of this effect remains unclear. We also encountered limitations due to missing data or rather our sample size, which prevented us from controlling for additional variables that may have contributed to the variance in the model (e.g., dementia severity, hospitalization of the PwD, multimorbidity of the IC, age of the IC, and type of relationship between IC and PwD). In addition, our study size may not have had adequate sensitivity to detect small effects. Future studies could likewise explore interaction effects to better understand the interplay between burden, resilience, and stress. Moreover, although we were able to identify some clear associations, we were limited to cross-sectional data, which prevents conclusions about causality. A minor limitation may be that we were unable to conduct an additional psychometric evaluation (in terms of reliability) of our validated instruments.

In conclusion, personal constraints in ICs may be linked to QoL in PwD, and high resilience may be particularly relevant in understanding IC stress. This knowledge could guide future interventions aimed at strengthening ICs and PwD. Special attention might be given to cohabiting dyads of ICs and PwD, as these relationships may present unique challenges and stressors that can be mitigated through targeted interventions. From a broader perspective, these results are in line with Pearlin et al.’s stress‑process framework [21], suggesting that IC stress is driven by the dynamic interplay of multiple stressors and resources.

## Data Availability

The study data will not be publicly available as it is potentially identifying/confidential patient data and, therefore, not compatible with the data protection concept of the study.

## Abbreviations

BIZA-D: Berlin Inventory on Relatives’ Burden in Dementia
BPSD: Dementia-specific behavioral disturbances
BRS: Brief Resilience Scale
CI: Confidence interval
IC: Informal caregivers
PSS: Perceived Stress Scale
PwD: People living with dementia
QoL: Quality of life
QoL-AD: Quality of Life in Alzheimer's Disease
VIF: variance inflation factor
